# The Social Distance Scale (v1): A Screening Instrument to Assess Patient Adherence to Prevention Strategies during Pandemics

**DOI:** 10.3390/ijerph17218158

**Published:** 2020-11-04

**Authors:** Michaela Prachthauser, Jeffrey E. Cassisi, Thien-An Le, Andel V. Nicasio

**Affiliations:** 1 Department of Psychology, University of Central Florida, 4111 Pictor Ln, Orlando, FL 32816, USA; prachthauser@knights.ucf.edu (M.P.); An.Le@knights.ucf.edu (T.-A.L.); 2 Department of Psychiatry and Behavioral Sciences, Medical University of South Carolina, 67 President Street, MSC 861, 4th Floor, IOP South, Charleston, SC 29425-8610, USA; andel.nicasio@gmail.com

**Keywords:** COVID-19, social distancing, pandemic, screening, prevention

## Abstract

Background: This paper describes the development of a brief self-report screening measure of adherence to social distancing and self-protective behaviors in pandemic situations. Items measured behaviors currently established as primary strategies to prevent and reduce the spread of the COVID-19 infection. Methods: An item pool of 29 questions was generated with the aim of estimating the frequency of specific behaviors and were written to avoid confounding the description of behavioral actions with evaluative judgements. Responses were collected from 401 young adults using an anonymous online survey. Results: An Exploratory Factor Analysis was conducted with the purpose of item reduction and subscale development. A 14-item Social Distance Scale emerged, consisting of four subscales: Isolation from Community (IC), Work from Home (WH), Family Contact (FC), and Protective Behaviors (PB). The initial psychometric evaluation of the scales indicated adequate internal consistency and test-retest reliability. (4) Conclusions: The Social Distance Scale (v1) is a promising new instrument which may be applied at the population or individual level. It may be used in conjunction with COVID-19 testing to measure interactions between social distancing factors and transmission. In addition, a reliable screening measure has utility for health service providers to assess patient risk and to provide educational/counseling.

## 1. Introduction

Early evidence suggests that COVID-19 is transmitted efficiently and sustainably in the general population [1]. It is thought to be primarily contracted from person-to-person through the spread of respiratory droplets of symptomatic and asymptomatic carriers [2]. Thus, most countries impacted by COVID-19 have implemented preventative measures, such as social distancing to contain and inhibit the rate of transmission and prevent overextension of the health care system. Social distancing is likely to be one of the most effective interventions in inhibiting the spread of the virus [3]. The Centers for Disease Control and Prevention (CDC) describes social distancing as keeping “at least 6 feet (1.82 m) from other people who are not from your household in both indoor and outdoor spaces” [4]. To further minimize the spread of respiratory droplets, the CDC also recommends that, at an individual level, social distancing should also be paired with other protective behaviors such as mask wearing, hand washing, avoiding large gatherings, and substituting physical social events with virtual forms of communication [4].

Referencing studies of previous outbreaks can be beneficial and lends credence to the efficacy of social distancing. For example, a narrative synthesis of one dozen modeling studies focused social distancing behaviors in relation to influenza spread “estimated that workplace social distancing measures alone produced a median reduction of 23% in the cumulative influenza attack rate in the general population” [5]. Research performed on archival data from 43 cities during the 1918 influenza pandemic has also revealed that large scale social distancing behaviors such as preventing public gatherings and quarantining the ill were effective in reducing the spread of the Spanish flu [6]. Specifically, earlier implementation of social distancing produced lower death rates and lower infection rates. The authors reported “a statistically significant association between increased duration of nonpharmaceutical interventions [social distancing] and a reduced total mortality burden (Spearman *r* = −0.39, *p* = 0.005)” [6].

In the early stages of the current pandemic, several projection models were published to demonstrate the potential health and cost benefits of social distancing [7,8,9]. For example, Ferguson and colleagues [7] published one of the first transmission models for the United States and United Kingdom. In their model, projections compared potential effects of (1) suppression, (2) mitigation (flattening the curve), or (3) uncontrolled (no response to the pandemic). For an uncontrolled epidemic, Ferguson et al. [7] predicted that critical care bed capacity would be exceeded as early as the second week in April 2020, with an intensive care unit (ICU) care bed demand over 30 times greater than the maximum supply in both countries. Their transmission models also predicted that an absence of control measures, such as social distancing, would yield a peak in mortality (daily deaths) occurring only after 3 months of the pandemic and that approximately 81% of Great Britain and the U.S. would be infected over the course of the pandemic.

As of Fall 2020, the research on the benefits of social distancing in the COVID-19 pandemic are echoing the earlier findings with H1N1 [8,9]. The implementation of social distancing measures taken by the government in South Korea in February and March 2020, including advising the public to stay at home and public messaging about personal protective behaviors such as hand washing, showed significant reduction in healthcare visits due to influenza-like illness as compared to previous flu seasons where social distancing procedures were not taken [10]. The data emerging from the United States are telling a similar story. A preliminary investigation conducted by VoPham et al. [11] examined patterns of de-identified smartphone GPS data nationwide to estimate county-level social distancing. More specifically, GPS data allowed for a comparison of activity prior to county-enforced stay-at-home orders and activity during the stay-at-home orders in 3054 counties across the U.S. Results of their examination revealed that a 35% increase in social distancing was associated with 29% reduction in COVID-19 incidence and a 35% reduction in COVID-19 mortality [11].

On a global level, one study was found to examine the quantifiable differences in transmission rates across areas with varying social distancing policies [12]. Personal mobility data and COVID-19 transmission data from 134 countries were examined, specifically using the 14 days prior to the implementation of social distancing measures and comparing the data collected for 21 days following the measures. Researchers found that nations with regional or national social distancing policies exhibited significantly larger reductions in individual mobility. Results also yielded a strong correlation between the decrease in mobility and the decrease in COVID-19 spreading, among those nations [12]. This is one of the first known studies specifically comparing the change in COVID-19 spread across areas, and specifically illustrates the efficacy of social distancing. Furthermore, these findings are consistent with conclusions from past outbreaks, such as H1N1 and Spanish Flu [5,6,8,9]. Taken together, social distancing and self-protective behaviors have the potential to inhibit the spread of infection. While adherence to such guidelines is paramount to delaying the spread and effects of COVID-19, it is inconsistently practiced at an individual level across the United States [13], placing more community and family members at high risk of exposure to the disease.

Likewise, risk behaviors are described as health behaviors that increase the likelihood of a variety of illness conditions and supports the relationship between behavior and health. Risky lifestyle behaviors are typically screened for in primary care settings, to facilitate the initiation of behavioral interventions to improve health outcomes [13]. Thus, early identification of such risk behaviors allows clinicians to efficiently address them and mitigate effects in a productive manner. Examples of risky lifestyle behaviors are cigarette smoking, alcohol misuse, physical inactivity, unhealthy diet, and nonadherence to seatbelt use; it is arguable that engaging in social distancing and self-protective behaviors are significant and should be also assessed during the COVID-19 pandemic.

Being at the front line of physical health concerns, primary care clinicians are uniquely able to quickly identify at-risk individuals using various brief screening measures. Patient responses on screening measures can then, be followed by counseling sessions or other brief interventions to fit the patient’s needs and prevent the development of larger health issues. Furthermore, given the pace of primary care settings, brief screening measures that are quick and accurate are optimal for assessing various constructs, such as alcohol use, depression, and anxiety, for example [14,15].

Despite the value of such practices there is currently a lack of brief self-report measures assessing adherence to social distancing and protective behaviors that meet psychometric standards. Thus, the purpose of this study is not only to develop such a screening measure, but also to provide an initial psychometric evaluation. This has the potential to be helpful both at the population and individual level. For example, such a measure could be utilized by researchers in conjunction with COVID-19 testing to better ascertain the impact and benefits that social distancing has on transmission and to identify which social distancing and self-protective behaviors are associated with the greatest risk reduction. Such research may be applied in the study of risk-factors among underserved and minority communities that are currently disproportionately affected by the pandemic. Moreover, the ability to quickly and reliably measure social distancing behaviors in a standardized format may present a helpful tool for healthcare workers to assess the behaviors of the patients they are seeing, which in turn could be helpful for designing behavioral interventions and counseling for high-risk individuals.

## 2. Methods and Materials

### 2.1. Measures

#### 2.1.1. Demographic Questions

Demographic information collected in this study included questions about age, gender, race/ethnicity, marital status, and current living situation.

#### 2.1.2. Initial Social Distance Item Pool

During March and April of 2020, the authors generated an item pool of 29 questions which asked about various social distancing and self-protective behaviors. These items were identified from review of CDC announcements and NIH National Institute of Allergy and Infectious Disease directives available at the time. Items were anchored on a five-point Likert scale scored from 0 to 4, with 0 indicating low levels of social distancing/protective behaviors and 4 indicating high levels of social distancing/protective behaviors. The items targeted multiple areas of social and physical distancing including protective behaviors such as wearing a mask, shopping in public, participating in small or large group activities, working outside of the home, using technology for social contact, physically interacting with family members of varying ages, visiting nursing homes, utilizing public transportation, and attending religious and/or funeral services in person. Effort was made to make the questions as descriptive as possible and included behavioral anchors for each item. Items were also written to avoid confounding questions with intent, motivation, or emotional reaction associated with the behaviors. The complete Social Distance Item Pool is displayed in English and Spanish in Appendix A.

#### 2.1.3. Pandemic Adverse Events Scale

During March and April 2020, the authors generated a list of yes/no questions reflecting the major events an individual could experience in response to the pandemic. Six items were generated. They include questions about unemployment, death of a friend or family member, use of COVID-19 testing, etc. The Pandemic Adverse Events Scale is displayed in English and Spanish in Appendix B.

#### 2.1.4. Validity Check Questions

Three questions were included to assess whether participants responded in a random or careless manner. For example, these questions asked respondents to indicate “no” to a yes/no question or to respond “false” to a true/false question and they were interspersed across the Demographics Questionnaire and the Social Distance Item Pool. Participants were removed if they endorsed any of the items in the random or careless direction.

### 2.2. Participants

Participants were undergraduate students enrolled in introductory psychology courses at a large university in the southeastern United States. They were recruited to participate in ongoing research monitoring social distancing behaviors and personal COVID-19 pandemic impact for course credit. Introductory psychology is a required course in the general education curriculum for most majors at this university. Therefore, the undergraduate population and all majors were well represented. Eligibility criteria required participants to be over the age of 18 and have the ability to complete an online questionnaire in the English language. The research study was exempted by the Institutional Review Board at the University of Central Florida since no protected health information (PHI) was collected.

### 2.3. Procedure

Participants logged into SONA (Sona Systems, Ltd., Trummi 5, 12616 Tallinn, Estonia; https://www.sona-systems.com), a cloud-based research software system for universities to ensure course credit is provided. SONA anonymously linked participants to Qualtrics which delivered the online survey measures and stored their responses. Initially, 579 participants completed the first wave of data collection. This data set was then reviewed for random, inconsistent, and incomplete responding. Participants with three or more item non-responses were eliminated (*n* = 120). Individuals who answered more than one validity check question incorrectly or who completed the same survey repeatedly in wave 1 were removed from the data set (*n* = 58). The resulting n was 401. The average age of participants in the first wave was 20.5 years with a SD = 4.8 years. The demographic description of the participants is listed in Table 1.

All statistical analyses were performed in SPSS (version 26, IBM, Armonk, NY, USA). The Social Distance Item Pool first underwent a series of Exploratory Factor Analyses (EFAs) with Principal Axis Factoring (PAF). This approach is recommended when there is little theoretical or a priori guidance available about the factor structure and the initial goal is to empirically summarize and identify latent dimensions within a data set [16,17]. This statistical approach is considered appropriate for the purposes of narrowing down a large pool of items to a smaller set of constructs or components and is consistent with the developmental nature of this study.

The goal of the second wave of data collection was to establish test–retest reliability of the resultant subscales. Initially, 168 participants from the first wave of data collection completed the measures again during the second wave. Participants were matched on SONA identification numbers which do not contain PHI. The same procedure was used for ensuring data quality in the second wave of data. Individuals who answered more than one validity check question incorrectly or who completed the same survey repeatedly in wave 2 were removed from the data set (*n* = 59). The resulting n for the Test–Retest Reliability analysis was 109. The average age of participants in the second wave was 20.3 years with a SD = 5.3 years.

The impact of the current pandemic on the participants was assessed with the Pandemic Adverse Events scale which lists common experiences as yes/no questions. Table 2 presents the participants’ responses to these questions. Unemployment of a household member was the most endorsed adverse event, where 45.9% of participants experienced this event.

Taken together, review of responses to the Pandemic Adverse Events Scale indicate that the sample of participants used in this study have been impacted by the current pandemic.

## 3. Results

### 3.1. Steps for Analysis

**Step 1**: Descriptive analyses were conducted on items to evaluate range, skewness, and kurtosis. As a result, items SD8 and SD10 were removed due to extreme violations of normality. Sample size was then evaluated. Multiple metrics for ascertaining the appropriateness of the size of a data set exist. One rule of thumb is to have a 10 to 1 ratio of respondents to items while other sources suggest a minimum requirement of 300 respondents [16,17]. The data set with 27 items met both metrics provided by extant guidelines [16,17]. Additionally, a Kaiser-Meyer-Olkin (KMO) index was also used to evaluate sample adequacy. The KMO should be at least 0.6 [16,17] and these are reported with the EFAs conducted below.

**Step 2**: A preliminary EFA using PAF was performed on the 27-item pool using an oblique rotational procedure (Direct Oblimin). This produced a weak factor correlation matrix which indicated that oblique rotational methods were not indicated [16].

Then, an EFA using PAF and an orthogonal (Varimax) rotational procedure was performed. The number of factors were not specified and eigenvalues above 1.0 were extracted. This analysis produced an eight-factor rotated solution. The communalities table was then examined and items with extracted communalities below 0.30 were removed (see Appendix C). This process led to the removal of six items; SD4, SD6, SD11, SD16, PIS3, and PIS6. The obtained KMO for this analysis was adequate at 0.753. Bartlett’s Test of Sphericity was significant (χ^2^ (351) = 2270.575, *p* < 0.05), indicating that correlations in the matrix are present and that the dimensions of the data set can be reduced.

**Step 3**: A third EFA using PAF and varimax rotation was conducted on the 21 retained items. The number of factors were not specified in this analysis and factors with eigenvalues above 1.0 were extracted. This analysis produced a seven-factor rotated solution. The communalities table was examined and item SD5 was removed due to having a loading below 0.30. Chi-squared goodness of fit tests were performed for solutions between one and seven factors and they were significant for the one- through six-factor solutions. The test for the seven-factor solution was non-significant (χ^2^ (71) = 87.5, *p* < 0.09) thus supporting a seven-factor solution.

**Step 4**: A fourth EFA using PAF and varimax rotation specifying seven factors was then performed on the 20-item pool, based on the previous results. Generally, three items per factor are needed to identify common factors [17]. Of the seven factors, three were removed due to an insufficient number of items loading on them (less than three). The remaining four factors were as follows: Factor one with items SD2, SD3, SD9, and PIS4; factor two with items SD7, SD13, SD14; factor three with items SD12, PIS7, PIS9, and PIS10; and factor four with items SD1, SD15, and SD17. Factors 5, 6, and 7 were the two item factors that were removed along with their associated items (SD15, SD17, PIS1, PIS2, PIS11, and PIS12).

**Figure 1:**
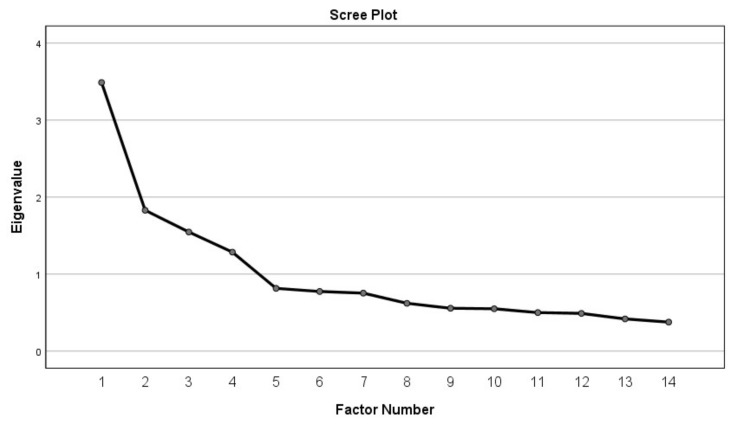
Scree Plot of Eigenvalues.

**Step 5**: A fifth EFA using PAF and varimax rotation was conducted on the retained 14 items. The number of factors were not specified in this analysis and factors with eigenvalues above 1.0 were extracted. This analysis produced a four-factor rotated solution (see Table 3). The obtained KMO for this analysis was adequate at 0.762. Bartlett’s Test of Sphericity was significant (χ^2^ (91) = 1226.302, *p* < 0.05). The scree plot produced by this analysis supported the use of a four-factor solution (see Figure 1). All 14 items loaded on the same four factors as they did in step 4.

**Step 6:** At this point the internal consistency of the four Factors was evaluated. Factor 1 had a Cronbach’s alpha of 0.717, Factor 2 had a Cronbach’s alpha of 0.669, Factor 3 had a Cronbach’s alpha of 0.656, and Factor 4 had a Cronbach’s alpha of 0.588. The internal consistency of factors was not improved with the removal of any item. Thus, the Cronbach’s alpha for the four factors were in the acceptable range.

**Step 7:** The items loading highly on each factor were reviewed to best characterize the factor interpretation and label. Indeed, the items that loaded together on each factor appeared to have face validity and related to similar constructs and behavioral patterns. The four items loading on Factor 1 included behaviors such as attending social gatherings and leaving the home to obtain groceries or medicine and was labeled Isolation from Community (IC). The three items loading on Factor 2 described an individual’s ability to work remotely and the overall level of social distancing at their workplace and was labeled Work from Home (WH). The three items loading on Factor 3 addressed contact with family members of different ages and family members with health concerns and was labeled Family Contact (FC). The three items loading on Factor 4 included behaviors such as mask wearing, hand washing, and maintaining a 6-foot distance from others and was labeled Protective Behaviors (PB). These four factors are treated as the four subscales of the Social Distance Scale, version 1 (SDS v1).

**Step 8***:* The test–retest reliability of the four factors (now subscales) was examined using the data collected from wave 2, the subsample of 109 participants who completed the survey twice, on average, 7 days apart. The results indicated good test–retest reliability for the four subscales. The test–retest correlations between the wave 1 and 2 administrations were 0.66, 0.80, 0.78 and 0.69 for the Isolation from Community (IC) behaviors subscale, Work from Home (WH) subscale, Family Contact (FC) subscale, and the Protective Behaviors (PB) subscale respectively.

### 3.2. Subscale Means and Intercorrelations

Instructions for scoring the four subscales are found in Table 4.

The theoretical range of scores for the different subscales are as follows: IC (0 to 16), WH (0 to 12), FC (0 to 16), and PB (0 to 12). The intercorrelations of the 4 subscales is displayed in Table 5. The means and standard deviations of the subscale totals by sex are displayed in Table 6.

## 4. Discussion

COVID-19 is currently a severe worldwide pandemic. The goal of this paper is to describe the development of a brief self-report screening measure of adherence to social distancing and self-protective behaviors in pandemic situations. We began by generating 29 items currently established as primary strategies to prevent and reduce the spread of the COVID-19 infection. This item pool underwent a series of EFAs for the purpose of item reduction and to identify the underlying constructs of social distancing and self-protective behaviors. A 14-item SDS (v1) that contained four subscales was refined. These four subscales represent constructs that are congruent with current theories about the behavioral and social risk factors for infection [18,19,20,21] that include community and family exposure, the inability to work from home, and lower adherence with self-protective behaviors. Initial means and SD of the summary scores for the four subscales is presented by gender for young adults in Table 5. At this time, this table may be used for interpretation with young adult college students between the ages of 18 and 25, in the United States, however additional normative work needs to be conducted before it may be used in Spanish, other age ranges, other socioeconomic groups, and internationally.

### 4.1. Limitations and Future Directions

One limitation of the current psychometric study is the tremendous situational variance associated with the pandemic. The data collection in this study took place in Florida during periods of both relatively low rates of infection and at a time of surge in the rates of infection. During this period, participants were transitioned from on campus instruction to remote learning. In additional to potential physical location changes experienced during data collection, information and recommendations disseminated about self-protection during the pandemic changed significantly over this study period. These mixed messages present a challenge to research on the prevention of COVID-19 [22]. More specifically, in March and April 2020 the CDC and several government representatives communicated several mixed messages, which may have discouraged the use of face masks and social distancing. Taken together, time 1 and time 2 responses may have been collected when perceptions of the importance of adherence differed greatly. These considerations, therefore, exemplify the potential impact of situational variance on increasing the error inherent in behaviorally anchored scales.

As implied in the discussion above, the generalizability of our initial results remains to be established. The development of any self-report scale requires multiple studies and replication. The current study justifies further efforts to continue this research. These results need to be replicated with more heterogenous populations across age ranges, socioeconomic groups, and various national/cultural regions. Additionally, construct validity must be demonstrated by establishing high correlations of the subscales with other measures they should correlate with such as cell phone location data (convergence), and low correlations with other measures they should not correlate with such as social desirability (divergence). Ultimately construct validity needs to be established with confirmatory factor analysis (CFA) using a large sample, most likely with pooled cross-sectional data from multiple locations.

The SDS (v1) is not intended to substitute for contact tracing. Many specific locations and situations are likely to be identified as spreader events through professional contact tracing. Such events are likely to lead to highly specific information which is difficult to categorize. For example, items such as SD8, SD11, and SD16 did not load on the current factors, and they included the use of public transportation, nursing home visits, and emergency room visits. Each of these areas are important to assess. They may or may not load on one of the factors with different samples in the future. To reiterate, the SDS (v1) is intended to be used as brief measure of adherence to behavioral prevention strategies during pandemics such as COVID-19 in both research and clinical screening contexts. Contact tracing has a different purpose and uses structured phone interviews.

The development of additional items for the SDS (v1) should be considered to enhance the three factors that were eliminated because of too few items. The eliminated factors related to emotional distancing, use of technology for social contact, and family illness. We have made the entire social distance item pool available in Appendix A because the scale may evolve with future research and with a more heterogeneous sample.

### 4.2. Clinical Implications

Future pandemics are likely to arise. Before treatment and vaccines can be developed, the initial response to these pandemics will likely rely social distancing and self-protective behaviors. The SDS (v1) was written to be useful in future situations calling for these types of prevention strategies. The utility of the scale needs to be established both for COVID-19 and other highly contagious respiratory infections.

We encourage the use of the SDS (v1) but urge caution in interpreting individual patient summary scores until additional normative data are collected and psychometrically evaluated. Ideally, initial use may simply involve reviewing items with the individual patient to identify potentially problematic areas. Results of such a review with the patient can inform healthcare recommendations and counseling in an educational and supportive context with them. In the meantime, we encourage collection of local norms for future use.

There is no cost for the use and dissemination of the SDS (v1). The question stems may be found on Table 3. The English and Spanish translations of the entire social distance item pool are presented in Appendix A. We recognize that additional research with the entire item pool and the development of additional items may be needed. Clearly one line of research that is needed is to demonstrate the equivalence of the English and Spanish forms of the questionnaire.

## 5. Conclusions

The Social Distance Scale (v1) is a brief self-report screening measure of patient adherence to social distancing and self-protective behaviors during the COVID-19 pandemic. While several scales are informally circulating at this stage of the pandemic, the Social Distance Scale (v1) now has initial psychometric information to support its use. Data from our study totaling 401 participants provide initial evidence for the factor structure and reliability of this measure. A common EFA indicated that 14 of the items loaded on 4 factors. These are considered the four subscales of the instrument. They are: Isolation from the Community (CI, four items), Work from Home (WH, three items), Family Contact (FC, four items), and Protective Behaviors (PB, three items). Lower scores indicate lower adherence or engagement with healthy behaviors and practices. Given that subscales did not correlate highly with each other, analyses support that the subscales measure different constructs. In addition, adequate internal consistency and test–retest reliability was demonstrated with the 4 subscales.

## Figures and Tables

**Table 1:** Participants’ gender ^1^, race, marital status, and college status.

Variables	Male (*n* = 124)		Female (*n* = 275)	
	*N*	%	*N*	%
Race				
White	91	22.8	199	49.8
Black	12	3.0	32	8.0
Asian/Pacific Islander	12	3.0	26	6.6
Native American	2	0.5	1	0.4
Other	6	1.5	17	4.3
Ethnicity				
Non-Hispanic/Latino	98	24.4	204	50.9
Hispanic/Latino	26	6.5	71	17.7
Marital status				
Not married	117	29.2	254	63.5
Married	7	1.7	21	5.2
College status				
Freshman	49	12	118	28.9
Sophomore	37	9	43	10.5
Junior	22	5.4	56	13.7
Senior	16	3.9	60	14.7
Unclassified	2	0.5	4	1

^1^ Two participants listed “other” for Gender; Note: Percentages may not equal 100 because of rounding error.

**Table 2:** Participants’ self-reported experience on the Pandemic Adverse Events Scale.

Question	Yes		No	
	*N*	%	*N*	%
Have one (or more members) in your household become (or remained) unemployed the past month?	184	45.9	217	54.1
Have there been any days the past month that you didn’t know where your next meal was coming from, or you involuntarily ate less than you needed?	39	9.7	362	90.3
Have you had a close friend or family member pass away in the past month?	39	9.7	362	90.3
Have you been tested for COVID-19?	59	14.7	342	85.3
(If you have been tested forCOVID-19, did you testpositive?) ^1^	6	10.2	45	76.3
Have there been barriers to obtaining the medicines you need the past month?	30	7.5	369	92.0
Have there been barriers for your receiving health care the past month?	64	16.0	337	84.4

^1^ Eight (13.6 percent) of people tested were not told the results; Note: Percentages may not equal 100 because of rounding error.

**Table 3:** Factor loadings Based on a Principal Axis Factoring and Varimax Rotation ^a^ for 14 items from the Social Distance Item Pool (*n* = 401).

	Factor			
	1	2	3	4
**SD1.** During the past month, I have stayed at least 6 feet away from other people when outside of my home:	0.370	0.189	0.048	**0.421**
**SD2.** During the past month, I have gone to small social gatherings with less than 10 people in public places, such as public parks or restaurants:	**0.683**	0.027	0.031	0.208
**SD3.** During the past month, I have gone to small social gatherings with less than 10 people in private places, such as my friend’s home:	**0.712**	0.034	0.052	0.042
**SD7.** During the past month, I have worked/studied from home:	−0.031	**0.532**	−0.003	0.247
**SD9.** During the past month, I have left my home to purchase gas, work, medicine, and groceries:	**0.458**	0.351	0.107	−0.003
**SD12.** During the past month, we have had small gatherings of family members at my place, or a relative’s home:	0.359	−0.038	**0.423**	−0.014
**SD13.** During the past month, I have been required to go to my place of employment, worksite, or school (away from home):	0.226	**0.855**	−0.062	−0.092
**SD14.** During the past month, I have been able to stay at least 6 feet away from other people when at my place of employment, worksite, or school:	0.139	**0.550**	0.153	0.290
**SD15.** During the past month, I have worn a face mask when I am in public, at my worksite, or school:	0.184	0.079	0.110	**0.619**
**SD17.** During the past month, when I am away from home, I have used hand sanitizer or washed my hands after I have touched objects such as doorknobs, computer keyboards, computer mice etc.	0.036	0.091	0.087	**0.550**
**PIS4.** I have been physically distant from others living outside of my home this past month:	**0.575**	0.171	0.102	0.192
**PIS7.** I have visited my elderly family members (who are 65 and up) this past month:	0.101	0.022	**0.522**	0.067
**PIS9.** I have visited with family members (64 and below) living outside of my home this past month:	0.108	0.004	**0.789**	0.086
**PIS10.** I have visited my family members who have serious health conditions this past month:	−0.071	0.065	**0.530**	0.100

^a^ Rotation converged in 5 iterations; Note: Item numbers and the highest factor loading are in bold.

**Table 4:** Scoring Instructions for the Social Distance Scale (v.1).

Isolation from Community (IC, four items)	Sum Items: SD2, SD3, SD9, PIS4
Work from Home (WH, three items)	Sum Items: SD7, SD13, SD14
Family Contact (FC, four items)	Sum Items: SD12, PIS7, PIS9, PIS10
Protective Behaviors (PB, three items)	Sum Items: SD1, SD15, SD17

**Table 5:** Intercorrelations Social Distance Scale (v1) subscale scores.

Variables	WH	FC	PB
IC	0.33	0.25	0.26
WH		0.10	0.24
FC			0.19

Note: CI = Isolation from Community subscale, WH = Work from Home subscale, FC = Family Contact subscale, PB = Protective Behaviors subscale.

**Table 6:** Participants’ Social Distance Scale (v1) subscale scores by gender ^1^.

Variables	Male (*n* = 124)		Female (*n* = 275)		Total (*n* = 401)	
	M	SD	M	SD	M	SD
IC	10.2	3.1	10.3	3.0	10.3	3.0
WH	9.5	2.9	9.7	2.8	9.6	2.8
FC	13.8	2.3	14.1	2.1	14.0	2.2
PB	10.0	1.9	10.7	1.5	10.5	1.7

^1^ Two participants listed “other” for gender; Note: CI = Isolation from Community subscale, WH = Work from Home subscale, FC = Family Contact subscale, PB = Protective Behaviors subscale.

## Appendix A

**Initial Social Distance Item Pool (v1, English)**

Check the one alternative that best describes your activities THE PAST MONTH

**SD1.** During the past month, I have stayed at least 6 feet away from other people when outside of my home:

- Never (0)
- Rarely (1)
- Sometimes (2)
- Often (3)
- Always (4)

**SD2.** During the past month, I have gone to small social gatherings with less than 10 people in public places, such as public parks or restaurants:

- Never (4)
- Once a week or less (3)
- 2–3 times a week (2)
- 4–6 times a week (1)
- Daily (0)

**SD3.** During the past month, I have gone to small social gatherings with less than 10 people in private places, such as my friend’s home:

- Never (4)
- Once a week or less (3)
- 2–3 times a week (2)
- 4–6 times a week (1)
- Daily (0)

**SD4.** During the past month, I have gone to crowded places and large gatherings of people, such as concerts and sporting events:

- Never (4)
- Once a week or less (3)
- 2–3 times a week (2)
- 4–6 times a week (1)
- Daily (0)

**SD5.** During the past month, I have shaken hands with people out of habit:

- Never (4)
- Rarely (3)
- Sometimes (2)
- Often (1)
- Always (0)

**SD6.** During the past month, I have used grocery/restaurant delivery services instead of going to the store:

- Never (0)
- Rarely (1)
- Sometimes (2)
- Often (3)
- Always (4)

**SD7.** During the past month, I have worked/studied from home:

- Never (0)
- Rarely (1)
- Sometimes (2)
- Often (3)
- Always (4)

**SD8.** During the past month, I have used public transportation (bus, subway, or train):

- Never (4)
- Rarely (3)
- Sometimes (2)
- Often (1)
- Always (0)

**SD9.** During the past month, I have left my home to purchase gas, work, medicine, and groceries:

- Never (4)
- Once a week or less (3)
- 2–3 times a week (2)
- 4–6 times a week (1)
- Daily (0)

**SD10.** During the past month, I have gone to a nursing home:

- Never (4)
- Once a week or less (3)
- 2–3 days a week (2)
- 4–6 days a week (1)
- Daily (0)

**SD11.** During the past month, I have gone to an emergency room or medical clinic:

- Never (4)
- Only once or twice (3)
- 3–5 times in the past month (2)
- Several times a week (1)
- Daily or almost daily (0)

**SD12.** During the past month, we have had small gatherings of family members at my place, or a relative’s home:

- Never (4)
- Once a week or less (3)
- 2–3 times a week (2)
- 4–6 times a week (1)
- Daily (0)

**SD13.** During the past month, I have been required to go to my place of employment, worksite, or school (away from home):

- Never (4)
- Once a week or less (3)
- 2–3 times a week (2)
- 4–6 times a week (1)
- Daily (0)

**SD14.** During the past month, I have been able to stay at least 6 feet away from other people when at my place of employment, worksite, or school:

- Never (0)
- Rarely (1)
- Sometimes (2)
- Often (3)
- Always, or I do not leave home for these activities (4)

**SD15.** During the past month, I have worn a face mask when I am in public, at my worksite, or school:

- Never (0)
- Rarely (1)
- Sometimes (2)
- Often (3)
- Always (4)

**SD16.** During the past month, I have worn disposable gloves when I am in public, at my worksite, or school:

- Never (0)
- Rarely (1)
- Sometimes (2)
- Often (3)
- Always (4)

**SD17.** During the past month, when I am away from home, I have used hand sanitizer or washed my hands after I have touched objects such as doorknobs, computer keyboards, computer mice etc.:

- Never (0)
- Rarely (1)
- Sometimes (2)
- Often (3)
- Always (4)

**PIS1.** I have had phone contact with others outside of my family this past month:

- Daily (4)
- 4–6 times a week (3)
- 2–3 times a week (2)
- Once a week (1)
- Never (0)

**PIS2.** I have had video contact with others outside of my family this past month:

- Daily (4)
- 4–6 times a week (3)
- 2–3 times a week (2)
- Once a week (1)
- Never (0)

**PIS3.** I have connected with others using social media this past month:

- Daily (4)
- 4–6 times a week (3)
- 2–3 times a week (2)
- Once a week (1)
- Never (0)

**PIS4.** I have been physically distant from others living outside of my home this past month:

- Always (4)
- Most of the time (3)
- About half the time (2)
- Sometimes (1)
- Never (0)

**PIS5.** I have been emotionally distant from others living outside of my home this past month:

- Always (4)
- Most of the time (3)
- About half the time (2)
- Sometimes (1)
- Never (0)

**PIS6.** I have attended religious services at my place of worship this past month:

- 4–6 times a week (0)
- 2–3 times a week (1)
- Once a week (2)
- Occasionally, but I stay in my car in the parking lot and 6 feet away from others (3)
- Never (4)

**PIS7.** I have visited my elderly family members (who are 65 and up) this past month:

- Daily (0)
- 4–6 times a week (1)
- 2–3 times a week (2)
- Once a week (3)
- Never (4)

**PIS8.** I have been emotionally distant from family members living outside of my home this past month:

- Always (4)
- Most of the time (3)
- About half the time (2)
- Sometimes (1)
- Never (0)

**PIS9.** I have visited with family members (64 and below) living outside of my home this past month:

- Daily (0)
- 4–6 times a week (1)
- 2–3 times a week (2)
- Once a week (3)
- Never (4)

**PIS10.** I have visited my family members who have serious health conditions this past month:

- Daily (0)
- 4–6 times a week (1)
- 2–3 times a week (2)
- Once a week (3)
- Never (4)

**PIS11.** I have cared for family members outside of my household who are ill this past month:

- Daily (0)
- 4–6 times a week (1)
- 2–3 times a week (2)
- Once a week (3)
- Never (4)

**PIS12.** I have attended funeral services this past month:

- Yes, I have gone to more than two at funeral halls or at places of worship (0)
- Yes, I have gone to one at a funeral hall or at a place of worship (1)
- Yes, I have gone to more than two, but I stayed outside, or in my car, and 6 feet away
- from others (2)
- Yes, I have gone to one, but I stayed outside, or in my car, and 6 feet away from others (3)
- No, or I viewed the online service for the person who passed (4)

**Grupo de Preguntas Iniciales de Distancia Social (v1, Español)**

Marque la alternativa que mejor describe sus actividades DURANTE EL MES PASADO:

**SD1s.** Durante el mes pasado, me he mantenido al menos a 6 pies de distancia de otras personas cuando estoy fuera de mi casa:

- Nunca (0)
- Raramente (1)
- A veces (2)
- A menudo (3)
- Siempre (4)

**SD2s.** Durante el mes pasado, asistí a pequeñas reuniones sociales con menos de 10 personas en lugares públicos, como parques públicos o restaurantes:

- Nunca (4)
- Una vez a la semana o menos (3)
- 2–3 veces a la semana (2)
- 4–6 veces por semana (1)
- Diario (0)

**SD3s.** Durante el mes pasado, asistí a pequeñas reuniones sociales con menos de 10 personas en lugares privados, como la casa de mi amigo/a:

- Nunca (4)
- Una vez a la semana o menos (3)
- 2–3 veces a la semana (2)
- 4–6 veces por semana (1)
- Diario (0)

**SD4s.** Durante el mes pasado, he ido a lugares concurridos y a grandes reuniones de personas, como conciertos y eventos deportivos:

- Nunca (4)
- Una vez a la semana o menos (3)
- 2–3 veces a la semana (2)
- 4–6 veces por semana (1)
- Diario (0)

**SD5s.** Durante el mes pasado, estreché la mano de personas por costumbre:

- Nunca (4)
- Raramente (3)
- A veces (2)
- A menudo (1)
- Siempre (0)

**SD6s.** Durante el mes pasado, utilicé servicios de entrega de compras de comida/restaurantes (por ej. Delivery) en lugar de ir de compras:

- Nunca (0)
- Raramente (1)
- A veces (2)
- A menudo (3)
- Siempre (4)

**SD7s.** Durante el mes pasado, he trabajado/estudiado desde casa:

- Nunca (0)
- Raramente (1)
- A veces (2)
- A menudo (3)
- Siempre (4)

**SD8s.** Durante el mes pasado, utilicé el transporte público (autobús, metro o tren):

- Nunca (4)
- Raramente (3)
- A veces (2)
- A menudo (1)
- Siempre (0)

**SD9s.** Durante el mes pasado, salí de mi casa para trabajar y para comprar gasolina, medicinas y comida:

- Nunca (4)
- Una vez a la semana o menos (3)
- 2–3 veces a la semana (2)
- 4–6 veces por semana (1)
- Diario (0)

**SD10s.** Durante el mes pasado, fui a un hogar de ancianos/as:

- Nunca (4)
- Una vez a la semana o menos (3)
- 2–3 veces a la semana (2)
- 4–6 veces por semana (1)
- Diario (0)

**SD11s.** Durante el mes pasado, fui a una sala de emergencias o clínica médica:

- Nunca (4)
- Solo una o dos veces (3)
- 3–5 veces en el último mes (2)
- Varias veces a la semana (1)
- Diario (0)

**SD12s.** Durante el mes pasado, hemos tenido pequeñas reuniones de familiares en mi casa o en la casa de un pariente:

- Nunca (4)
- Una vez a la semana o menos (3)
- 2–3 veces a la semana (2)
- 4–6 veces por semana (1)
- Diario (0)

**SD13s.** Durante el mes pasado, se me solicitó que fuera a mi lugar de trabajo o escuela (fuera de casa):

- Nunca (4)
- Una vez a la semana o menos (3)
- 2–3 veces a la semana (2)
- 4–6 veces por semana (1)
- Diario (0)

**SD14s.** Durante el mes pasado, pude mantenerme al menos a 6 pies de distancia de otras personas en mi lugar de trabajo o escuela:

- Nunca (0)
- Raramente (1)
- A veces (2)
- A menudo (3)
- Siempre, o no salgo de casa para estas actividades (4)

**SD15s.** Durante el mes pasado, he usado una máscara de protección cuando estoy en público, en mi lugar de trabajo o en la escuela:

- Nunca (0)
- Raramente (1)
- A veces (2)
- A menudo (3)
- Siempre (4)

**SD16s.** Durante el mes pasado, he usado guantes desechables cuando estoy en público, en mi lugar de trabajo o en la escuela:

- Nunca (0)
- Raramente (1)
- A veces (2)
- A menudo (3)
- Siempre (4)

**SD17s.** Durante el mes pasado, cuando estoy fuera de casa, he usado desinfectante para manos o me lavé las manos después de tocar objetos como puños de puertas, teclados de computadora, ratones, etc.

- Nunca (4)
- Raramente (3)
- A veces (2)
- A menudo (1)
- Siempre (0)

**PIS1s.** En el mes pasado, he tenido contacto por teléfono con otras personas fuera de mi familia:

- Diario (4)
- 4–6 veces por semana (3)
- 2–3 veces a la semana (2)
- Una vez a la semana (1)
- Nunca (0)

**PIS2s.** En el mes pasado, he tenido contacto por video con otras personas fuera de mi familia:

- Diario (4)
- 4–6 veces por semana (3)
- 2–3 veces a la semana (2)
- Una vez a la semana (1)
- Nunca (0)

**PIS3s.** En el mes pasado, me he conectado con otras personas usando las redes sociales:

- Diario (0)
- 4–6 veces por semana (1)
- 2–3 veces a la semana (2)
- Una vez a la semana (3)
- Nunca (4)

**PIS4s.** En el mes pasado, he estado físicamente distante de otras personas que viven fuera de mi casa:

- Siempre (4)
- La mayor parte del tiempo (3)
- Más o menos la mitad del tiempo (2)
- A veces (1)
- Nunca (0)

**PIS5s.** En el mes pasado, he estado emocionalmente distante de otras personas que viven fuera de mi casa:

- Siempre (4)
- La mayor parte del tiempo (3)
- Más o menos la mitad del tiempo (2)
- A veces (1)
- Nunca (0)

**PIS6s.** En el mes pasado, he asistido a servicios religiosos donde regularmente voy:

- 4–6 veces por semana (0)
- 2–3 veces a la semana (1)
- Una vez a la semana (2)
- De vez en cuando, pero me quedo en mi automóvil en el estacionamiento y a 6 pies de
- distancia de los demás (3)
- Nunca (4)

**PIS7s.** En el mes pasado, he visitado a mis familiares mayores (que tienen 65 años o más):

- Diario (0)
- 4–6 veces por semana (1)
- 2–3 veces a la semana (2)
- Una vez a la semana (3)
- Nunca (4)

**PIS8s.** En el mes pasado, he estado emocionalmente distante de los miembros de la familia que viven fuera de mi casa:

- Siempre (4)
- La mayor parte del tiempo (3)
- Más o menos la mitad del tiempo (2)
- A veces (1)
- Nunca (0)

**PIS9s.** En el mes pasado, he visitado a miembros de mi familia (de 64 años o menos) que viven fuera de mi casa:

- Diario (0)
- 4–6 veces por semana (1)
- 2–3 veces a la semana (2)
- Una vez a la semana (3)
- Nunca (4)

**PIS10s.** En el mes pasado, he visitado a los miembros de mi familia que tienen problemas de salud graves:

- Diario (0)
- 4–6 veces por semana (1)
- 2–3 veces a la semana (2)
- Una vez a la semana (3)
- Nunca (4)

**PIS11s.** En el mes pasado, cuidé de miembros de mi familia que estuvieron enfermos fuera de mi hogar:

- Diario (0)
- 4–6 veces por semana (1)
- 2–3 veces a la semana (2)
- Una vez a la semana (3)
- Nunca (4)

**PIS12s.** En el mes pasado, he asistido a servicios funerarios:

- Sí, he ido a más de dos en las salas funerarias o en los lugares de culto (0)
- Sí, he ido a uno en una sala funeraria o en un lugar de culto (1)
- Sí, he ido a más de dos, pero me quedé afuera, o en mi automóvil, y a 6 pies de distancia
- de los demás (2)
- Sí, he ido a uno, pero me quedé afuera, o en mi automóvil, y a 6 pies de distancia de los
- demás (3)
- No, o vi el servicio de la persona fallecida en linea (4)

## Appendix B

**Pandemic Adverse Events Scale (v1, English)**

Please make all ratings for the PAST MONTH

**PIS13.** Have one (or more members) in your household become (or remained) unemployed the past month:

- Yes (1)
- No (0)

**PIS14.** Have there been any days the past month that you didn’t know where your next meal was coming from, or you involuntarily ate less than you needed?

- Yes (1)
- No (0)

**PIS15.** Have you had a close friend or family member pass away in the past month?

- Yes (1)
- No (0)

**PIS16.** Have you been tested for COVID-19?

- Yes (1)
- No (0)

(Display This Question if PIS16 is Yes)

**PIS16a.** If you have been tested for COVID-19, did you test positive?

- Yes (1)
- No (0)
- I haven’t been told the results (1)

**PIS17.** Have there been barriers for your receiving health care the past month?

- Yes (1)
- No (0)

**PIS18.** Have there been barriers to obtaining the medicines you need the past month?

- Yes (1)
- No (0)

**Escala de Eventos Adversos de la Pandemia (v1, Español)**

Por favor conteste todas las oraciones en base al MES PASADO

**PIS13s.** Uno (o más miembros) en su hogar quedó (o permaneció) desempleado el mes pasado:

- Sí (1)
- No (0)

**PIS14s.** ¿Ha habido algún día en el mes pasado en el que no sabía de dónde vendría su próxima comida, o involuntariamente comió menos de lo que necesitaba?

- Sí (1)
- No (0)

**PIS15s.** ¿Ha fallecido un familiar o un amigo cercano en el último mes?

- Sí (1)
- No (0)

**PIS16s.** ¿Le han hecho la prueba de COVID-19?

- Sí (1)
- No (0)

**PIS17s.** ¿Ha habido barreras para obtener los medicamentos que necesita el mes pasado?

- Sí (1)
- No (0)

**PIS18s.** ¿Ha habido barreras para usted recibir atención médica el mes pasado?

- Sí (1)
- No (0)

## Appendix C

Communalities table from the second Exploratory Factor Analysis using Principal Axis Factoring and Varimax rotation for the 27 items retained from the Social Distance Item Pool (described in Step 2 of Results).

	**Initial**	**Extraction**
**SD1.** During the past month, I have stayed at least 6 feet away from other people when outside of my home:	0.343	0.408
**SD2.** During the past month, I have gone to small social gatherings with less than 10 people in public places, such as public parks or restaurants:	0.438	0.555
**SD3.** During the past month, I have gone to small social gatherings with less than 10 people in private places, such as my friend’s home:	0.398	0.491
**SD4.** During the past month, I have gone to crowded places and large gatherings of people, such as concerts and sporting events:	0.225	0.236
**SD5.** During the past month, I have shaken hands with people out of habit:	0.265	0.326
**SD6.** During the past month, I have used grocery/restaurant delivery services instead of going to the store:	0.120	0.104
**SD7.** During the past month, I have worked/studied from home:	0.305	0.363
**SD9.** During the past month, I have left my home to purchase gas, work, medicine, and groceries:	0.372	0.387
**SD11.** During the past month, I have gone to an emergency room or medical clinic:	0.092	0.126
**SD12.** During the past month, we have had small gatherings of family members at my place, or a relative’s home:	0.280	0.341
**SD13.** During the past month, I have been required to go to my place of employment, worksite, or school (away from home):	0.437	0.727
**SD14.** During the past month, I have been able to stay at least 6 feet away from other people when at my place of employment, worksite, or school:	0.398	0.494
**SD15.** During the past month, I have worn a face mask when I am in public, at my worksite, or school:	0.307	0.440
**SD16.** During the past month, I have worn disposable gloves when I am in public, at my worksite, or school:	0.164	0.222
**SD17.** During the past month, when I am away from home, I have used hand sanitizer or washed my hands after I have touched objects such as doorknobs, computer keyboards, computer mice etc.:	0.245	0.368
**PIS1.** I have had phone contact with others outside of my family this past month:	0.343	0.588
**PIS2.** I have had video contact with others outside of my family this past month:	0.339	0.482
**PIS3.** I have connected with others using social media this past month:	0.223	0.257
**PIS4.** I have been physically distant from others living outside of my home this past month:	0.404	0.568
**PIS5.** I have been emotionally distant from others living outside of my home this past month:	0.407	0.656
**PIS6.** I have attended religious services at my place of worship this past month:	0.186	0.177
**PIS7.** I have visited my elderly family members (who are 65 and up) this past month:	0.272	0.343
**PIS8.** I have been emotionally distant from family members living outside of my home this past month:	0.352	0.554
**PIS9.** I have visited with family members (64 and below) living outside of my home this past month:	0.387	0.563
**PIS10.** I have visited my family members who have serious health conditions this past month:	0.302	0.355
**PIS11.** I have cared for family members outside of my household who are ill this past month:	0.293	0.397
**PIS12.** I have attended funeral services this past month:	0.226	0.345
